# Healthcare Outcomes of Patients and Antecedents via Teleophthalmology in Eastern Taiwan during COVID-19

**DOI:** 10.3390/healthcare12161672

**Published:** 2024-08-21

**Authors:** Hsing-Chu Chen, Ching-Yu Chang, Chung-Hung Tsai, Wei-Lin Hsu, Wen-Fang Sung, Yu-Xuan Wu

**Affiliations:** 1 Head Office, Hualien Tzu Chi Hospital, Buddhist Tzu Chi Medical Foundation, Hualien 970, Taiwan; tz1049@tzuchi.com.tw; 2 Department of Public Health, Tzu Chi University, Hualien 970, Taiwan; 3 Medical Affairs Office, Hualien Tzu Chi Hospital, Buddhist Tzu Chi Medical Foundation, Hualien 970, Taiwan; jenny_chang@tzuchi.com.tw (C.-Y.C.); song0626@tzuchi.com.tw (W.-F.S.); jieru925@tzuchi.com.tw (Y.-X.W.); 4 Department of Information Technology and Management, Tzu Chi University, Hualien 970, Taiwan; wlhsu@gms.tcu.edu.tw

**Keywords:** telemedicine, teleophthalmology, healthcare accessibility, communication quality, service quality

## Abstract

Objective: The coronavirus disease 2019 (COVID-19) pandemic has caused significant transformations in healthcare. Many countries began the rapid development and adoption of telemedicine to avoid the spread of the pandemic and created an innovative model for healthcare delivery. This study identified the critical antecedents that affected the **considered** healthcare outcomes via **teleophthalmology** in Eastern Taiwan during the COVID-19 pandemic. Methods: This study’s participants included residents of five towns in Taitung County who had experience with teleophthalmology. This study analyzed the structured questionnaires completed by the participants to validate the proposed research framework. Statistical methods were used to verify the research models, including descriptive statistical analysis, confirmatory factor analysis, and structural equation modeling. The date of this study was from 1 October 2020 to 31 July 2023. Results: The results of this study reveal that the average monthly use of teleophthalmology by individuals in rural areas increased annually. Females tended to utilize teleophthalmology services more than males. There were no significant differences across any of the constructs with respect to age or educational level. Additionally, the patients’ awareness of healthcare accessibility via and the communication quality of teleophthalmology simultaneously affected teleophthalmology’s adoption and service quality, which in turn jointly affected health outcomes. Both healthcare accessibility and communication quality were the antecedents of the healthcare outcomes. The health outcomes refer to the impact of teleophthalmology on the quality of the patients’ health and well-being. Additionally, teleophthalmology’s adoption and service quality acted as mediators. Conclusions: This study’s findings are expected to increase attention to the healthcare outcomes and antecedents of teleophthalmology to promote better telemedicine practices and services for rural residents.

## 1. Introduction

The coronavirus disease 2019 (COVID-19) outbreak produced several challenges for healthcare services worldwide. In January 2020, the World Health Organization (WHO) declared the COVID-19 outbreak a Public Health Emergency of International Concern, followed by the declaration of a global pandemic in March 2020. Also, the WHO suggested the implementation of preventive measures, such as vaccination, social distancing, wearing masks, and maintaining good personal hygiene [1]. According to Patel et al. [2], although healthcare providers sought to protect their and their patients’ health, it was challenging to provide safe, timely, and effective healthcare delivery. Additionally, the COVID-19 pandemic accelerated the digital transformation and innovation of healthcare services, including innovations in the path of care and the technology used for diagnosis. Telemedicine emerged as a new digital health service [3].

Telemedicine is defined as the use of information and communication technology (ICT) by healthcare professionals to deliver healthcare services from a distance. Physically separating physicians and patients during a global pandemic protected both from the risk of infection [4,5]. Telemedicine is classified into two types: asynchronous and synchronous telemedicine. The first type is also called store-and-forward telemedicine, while the second type is called real-time telemedicine. Asynchronous telemedicine records information in advance and includes the exchange of the recorded information between two or more individuals at different times. In contrast, synchronous telemedicine is the real-time exchange of information between a healthcare worker and a patient [6,7].

Teleophthalmology is the application of telemedicine in the field of ophthalmology. Teleophthalmology has recently become widely accepted and is more frequently used in asynchronous telemedicine [8]. Synchronous telemedicine with remote monitoring and real-time interaction has also developed. The changes in health insurance coverage systems for telemedicine and its announcement and suggestion by international organizations have been significant factors in the rapid promotion of teleophthalmology [9].

This study aimed to focus on Tzu Chi Hospital Medical Center, the only medical center in Eastern Taiwan, to identify the critical antecedents that influenced healthcare outcomes via teleophthalmology in Eastern Taiwan during the COVID-19 pandemic. The research subjects were the patients of the eye clinic who received a diagnosis by teleophthalmology. Within the proposed research framework, both healthcare accessibility and communication quality were identified as the key antecedents of healthcare outcomes. The health outcomes were defined as the impact of teleophthalmology on the quality of the patients’ health and well-being [10]. A literature review and the research hypotheses are presented below.

### 1.1. Healthcare Accessibility

Enhancing healthcare accessibility can help people access the proper healthcare resources for maintaining and improving their health [11,12]. Healthcare accessibility has been defined as the timely adoption of personal health services to achieve the best outcomes for health [13]. Oginni et al. [14] suggested that access to healthcare involves the following five dimensions: availability, accessibility, accommodation, affordability, and acceptability. Providing individuals with timely and appropriate accessibility to healthcare providers and medical resources is crucial for monitoring, preventing, and maintaining overall health and welfare [15]. Therefore, many healthcare institutions promoted telemedicine during the COVID-19 pandemic as an alternative to face-to-face care for chronic diseases [16].

Many empirical studies have shown that patients’ awareness of healthcare accessibility via telemedicine positively affects telemedicine’s adoption. Papachristou et al. [17] indicated that healthcare accessibility is an essential factor for telemedicine acceptance. Haun et al. [18] found that teleconsultations enhance healthcare accessibility, which in turn enhances patients’ positive experiences of telemedicine. Li et al. [19] also found that if patients perceive the accessibility of health services as being time- and labor-saving, they have a more positive attitude toward participating in telemedicine. Landi et al. [20] found that the time-saving aspect of telemedicine is its most significant advantage. Thus, if patients feel that healthcare accessibility has increased via teleophthalmology, teleophthalmology adoption can be promoted. Therefore, this study proposes the following hypothesis:

**Hypothesis 1.**  *Healthcare accessibility has a positive impact on teleophthalmology adoption.*

The service quality of an information system (IS) refers to the overall support for the services delivered by an information system provider [21,22,23]. Some studies have used the SERVQUAL instrument to measure IS service quality [24]. The SERVQUAL instrument was developed by Parasuraman et al. [25] to measure service quality. The SERVQUAL instrument has been widely adopted in many fields and includes five dimensions of service quality: tangibility, reliability, responsiveness, assurance, and empathy [26]. Teleophthalmology is an innovative example of an online information system. Therefore, the service quality of telemedicine can be defined as the degree to which patients assess overall support for healthcare services provided by telemedicine providers [27,28].

Previous studies have shown that healthcare accessibility positively affects service quality. Anawade et al. [11] found that healthcare accessibility is a key component of healthcare service quality. Using in-depth individual and focus group interviews, Mosadeghrad [29] confirmed that accessibility affects healthcare service quality. Shafii et al. [30] verified that healthcare accessibility could affect service quality. Almuhanadi et al. [31] demonstrated that access to general medicine is an essential dimension of service quality.

Fatehi et al. [32] found that teleophthalmology can improve healthcare accessibility, which in turn makes services cost-effective in rural areas. Kludacz-Alessandri et al. [33] showed that healthcare accessibility through telemedicine improves the telemedicine service quality in primary medical care. Hence, improved healthcare accessibility via teleophthalmology among patients should positively influence service quality. Therefore, this study proposes the following hypothesis:

**Hypothesis 2.**  *Healthcare accessibility has a positive impact on service quality.*

### 1.2. Communication Quality

Unal et al. [34] argued that physician–patient communication is the communication pattern between healthcare service users and physicians. Good physician–patient communication can provide many medical benefits [35]. Sharkiya [36] demonstrated that communication functions affect health outcomes. Additionally, many studies have indicated that improved communication quality promotes better patient adherence and loyalty [34,37,38].

Hamlin et al. [39] showed that individuals generally have a positive attitude toward healthcare through remote online communication. Furthermore, recent research has shown that patients tend to follow their original treatment schedules after receiving telemedicine [40]. Sabetian et al. [41] showed that telemedicine is an effective tool for improving physician–patient communication. Previous studies have indicated that an online platform could provide channels for interaction and communication [42] and that better online interactivity and communication could improve online service adoption [43]. Xuan et al. [44] also proved that online physician–patient interpersonal communication positively influences online physician services of patient adoption. Therefore, this study proposes the following hypothesis:

**Hypothesis 3.**  *Communication quality has a positive impact on teleophthalmology adoption.*

The quality of the physician–patient communication provided via a telemedicine platform affects the quality of care. Gessesse et al. [37] demonstrated that physician–patient communication quality is vital for patients’ physical and mental welfare, including healthcare service quality. According to a previous study, improving the quality of the communication provided through the telemedicine platform enhances its service quality [45]. Parmanto et al. [46] showed that the quality of the communication provided via a telemedicine platform affects the perceived service quality. Salam and Bajaba [47] also indicated that the quality of the interaction between healthcare service providers and patients affects the service quality of the COVID-19 healthcare system. Therefore, patients’ awareness of communication quality via teleophthalmology can positively affect service quality. Thus, this study proposes the following hypothesis:

**Hypothesis 4.**  *Communication quality has a positive impact on service quality.*

### 1.3. Health Outcomes

The health outcomes mentioned in this study refer to the impact of telemedicine on the quality of patients’ health and well-being [10]. Telemedicine uses ICT and efficiently delivers healthcare services to remote patients using a digital approach [48]. According to DeLone and McLean Information Systems Success Model (D&M IS Success Model), the degree of telemedicine system adoption affects individuals’ health benefits [21,22]. The D&M IS Success Model has been regarded by many scholars as one of the most influential theories for explaining information systems success [47]. Research has revealed that patients are highly satisfied with the quality of the care provided by telemedicine [49]. Additionally, many studies have found that adopting a telemedicine platform affects patients’ self-care effectiveness, satisfaction, and perceived health outcomes [50,51]. Specifically, the increased use of teleophthalmology will offer better healthcare services for patients, thereby improving their health outcomes. Therefore, this study proposes the following hypothesis:

**Hypothesis 5.**  *Teleophthalmology adoption has a positive impact on health outcomes.*

Lokantari and Kristaung [45] proved that telemedicine’s service quality significantly and positively affects user satisfaction. Tantarto et al. [52] demonstrated that telemedicine’s service quality affects patients’ satisfaction. Moreover, according to D&M IS Success Model, telemedicine service quality influences user satisfaction, which in turn affects individual health benefits [21,22]. Salam and Bajaba [47] suggested that service quality regarding the COVID-19 healthcare system significantly influences system satisfaction and quality of life. Therefore, patients’ awareness regarding the service quality of teleophthalmology systems affects their health outcomes. Thus, this study proposes the following hypothesis:

**Hypothesis 6.**  *Service quality has a positive impact on health outcomes.*

**Figure 1:**
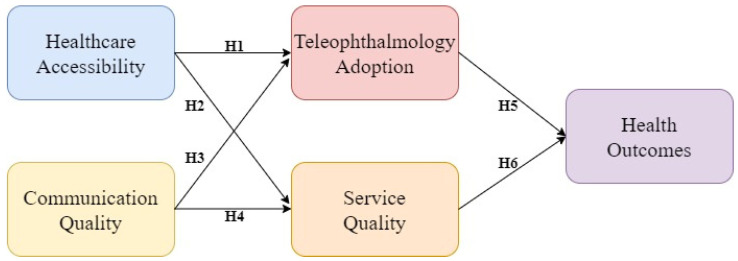
The proposed research framework.

In summary, Figure 1 shows the research framework proposed in this study after the literature review and hypothesis formation.

## 2. Materials and Methods

### 2.1. Implementation of Teleophthalmology in Eastern Taiwan

Taiwan’s Tzu Chi Medical Center initiated the “Telemedicine Specialist Diagnosis” project on 18 May 2020, to provide telemedicine to the residents of four rural towns in Taitung County, including Guanshan Township, Chishang Township, Luye Township, and Haiduan Township. Another town, Yanping Township, was included in April 2022. The consultation was conducted through a “three-party joint teleconsultation” (the specialists at Hualien’s Tzu Chi Medical Center, patients in Taitung County, and rural doctors in Taitung County). Starting from the first phase of the “Telemedicine Specialist Diagnosis” project, subsequent phases involved conducting surveys and research. The date of the study was from 1 October 2020 to 31 July 2023.

**Figure 2:**
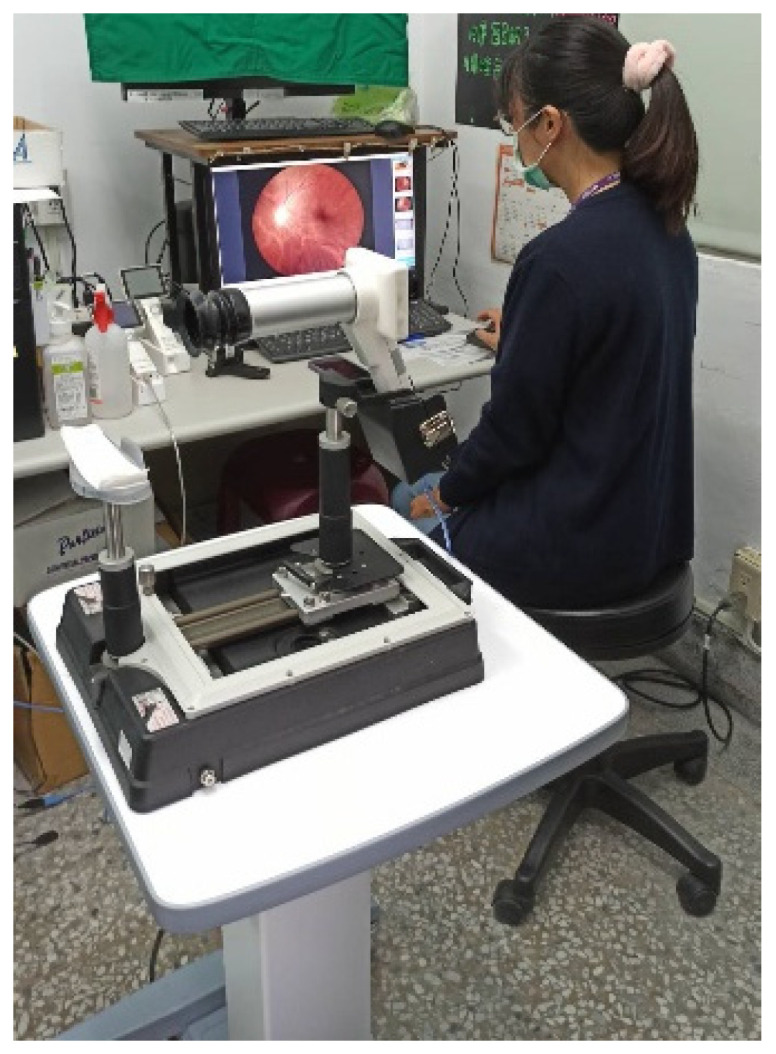
Tonometer.

**Figure 3:**
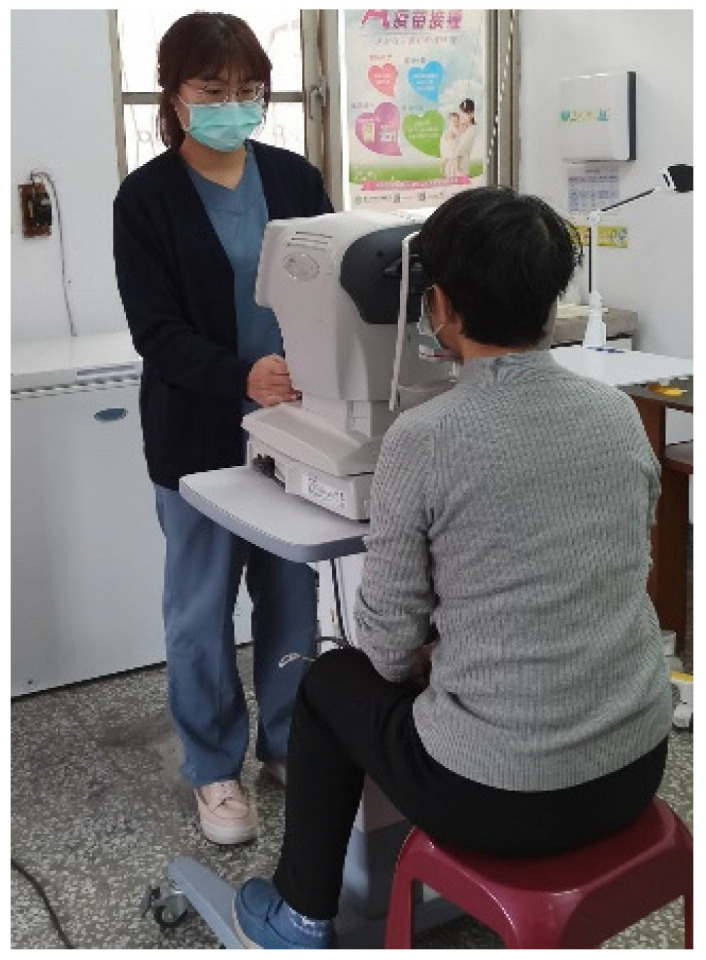
Slit lamp and fundus camera.

The main equipment for teleophthalmology includes the 5G network to connect with the medical center and rural health stations, a video conferencing system, and health information system (HIS) in the health station. The network and equipment can offer real-time audio and video interaction and communication, store electronic medical records, and transmit and exchange eye examination images. The optical instruments required for eye examination include tonometer, slit lamp, and fundus camera, as shown in Figure 2 and Figure 3.

**Figure 4:**
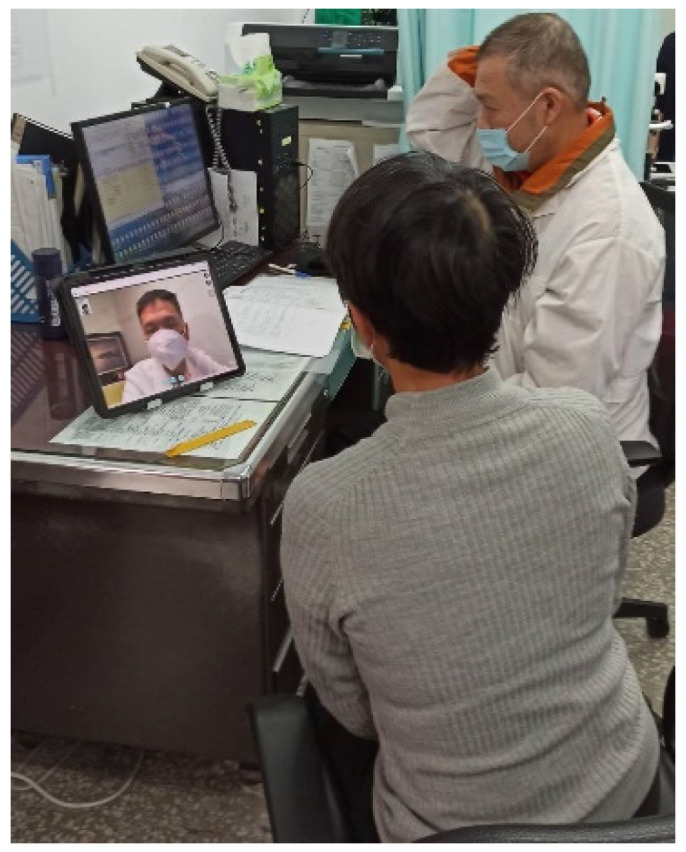
Teleconsultation: health station site.

**Figure 5:**
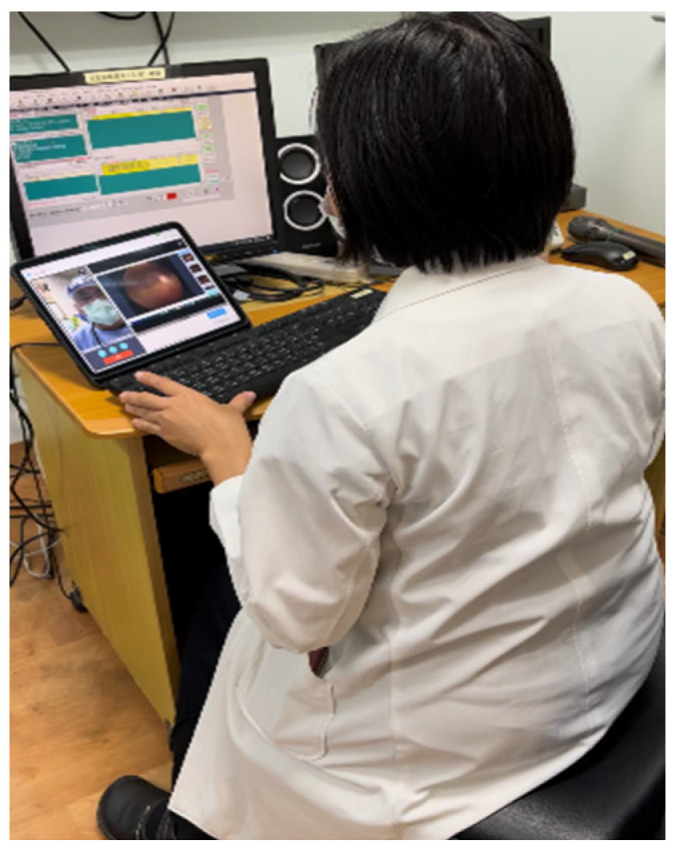
Teleconsultation: medical center site.

**Figure 6:**
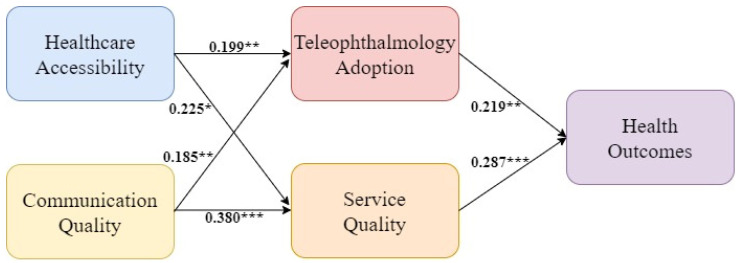
Final research framework. * *p* value < 0.05, ** *p* value < 0.01, *** *p* value < 0.001.

The process of consultation for teleophthalmology is (1) the personnel at the health station informs patients the date and time for teleconsultation in advance as well as helps them make an appointment; (2) on the day of the appointment, the nurse at the health station uses a tonometer to measure patients’ vision and intraocular pressure (Figure 2) and takes images with a slit lamp and fundus camera (Figure 3); (3) the doctor at the health station makes an inquiry and connects with the medical center for teleophthalmology (Figure 4); (4) the doctor at the health station shares the patient’s basic physiological information as well as the slip lamp and fundus camera images with the specialist at the medical center for diagnosis (Figure 5); (5) the specialist at the medical center starts the teleconsultation by video conferencing system with the patient (Figure 5); (6) the specialist at the medical center informs the patient the result of diagnosis and asks the doctor at the health station to prescribe medication (Figure 5); (7) virtual visit is completed, and the remote joint teleconsultation ends.

### 2.2. Research Population and Design

This cross-sectional study evaluated the implementation outcomes of teleophthalmology in Eastern Taiwan using a questionnaire survey. The study was conducted by Taiwan’s Hualien Tzu Chi Medical Center. The cross-sectional face-to-face questionnaires were administered during the COVID-19 pandemic between 1 October 2020 and 30 September 2021. The research population included the eye clinic patients in the five rural towns in Taitung County who had telemedicine experience. The Research Ethics Committee of Hualien Tzu Chi Medical Center approved this study on 30 September 2020.

### 2.3. Data Collection and the Establishment of the Scale

The questionnaire was divided into two parts. Part 1 comprised the demographic characteristics of the participants, including sex, age, educational level, place of residence, source of information for telemedicine, and critical diseases (such as diabetes, hypertension, and heart disease). Part 2 comprised questions on each research construct, including healthcare accessibility, communication quality, teleophthalmology adoption, service quality, and health outcomes. These items were rated on a five-point Likert Scale. Two items for healthcare accessibility were revised from the perceived usefulness scale developed by Alexandra et al. [4]. Two items for communication quality were revised from the interaction quality scale developed by Parmanto et al. [46]. Two items for teleophthalmology adoption were revised from the satisfaction and future use scale developed by Parmanto et al. [46]. Four items measuring service quality were revised from the teleophthalmology encounter quality scale developed by LeRouge et al. [53]. Two items for health outcomes were revised from the achieving success and quality of health life scale developed by [54,55,56] (Appendix A: Questionnaire Items).

Before finalizing the questionnaire, the draft was reviewed by experts and scholars in the professional areas of public health and teleophthalmology to establish face validity. Next, a pilot test was conducted with 30 participants. Questionnaires were formally issued after completing the pilot test. Written informed consent was obtained from the participants before administering the questionnaire.

The distribution of the questionnaire in this study was conducted by interviewers who visited the health stations in remote townships to conduct face-to-face interviews with patients. During the interview process, if participants have any questions about the questionnaire items, the interviewers will provide immediate assistance, explain the meaning of the items, and answer questions. Therefore, participants can understand and distinguish the differences in wording regarding the dimensions and items of the questionnaire.

### 2.4. Statistical Analysis

Statistical analysis consisted of two parts. Descriptive statistics and reliability analyses were performed on the demographic variables. Statistical analyses were performed using SPSS 28.0 software. Additionally, causal relationships among the five constructs of the scale were examined. Confirmatory factor analysis and structural equation modeling (SEM) were conducted using the AMOS 28.0 software. Additionally, a common method bias analysis was conducted. According to Podsakoff et al. [57], Harman’s single-factor test is widely used to determine whether common method bias is involved in the research data. Exploratory factor analysis was performed on all the items, and the results of unrotated factor analysis were reviewed to confirm the number of factors that were sufficient to explain the variance in the items. After verification, the first extracted factor explained 32.33% of the variance, which was less than 50% and did not present severe threats. In addition, a two-phase method was used to verify the model’s fit [58]. The measurement model was analyzed before the structural model was used. 

## 3. Results

From May 2020 to December 2022, the average monthly cases of outpatient visits for the teleophthalmology implemented by Tzu Chi Medical Center in the five townships are shown in Table 1. Generally, the average number of residents in rural areas using teleophthalmology increased yearly. This was particularly evident in Luye and Guanshan townships, where the average monthly visits increased yearly. This indicates that the residents of these two towns showed a higher acceptance of teleophthalmology.

In total, 181 participants answered the questionnaire, 15 of whom did not complete it or provide regular answers. After excluding the invalid questionnaires, 166 valid questionnaires were considered for analysis. Of the 166 participants, 104 were women, and 62 were men. Regarding age, the highest proportion of participants were aged between 61 and 70 years (*n* = 47, 28.3%), followed by aged between 71 and 80 years (*n* = 46, 27.7%). Regarding educational level, the highest proportion of participants had an elementary school education (*n* = 66, 39.8%), followed by illiteracy (*n* = 34, 20.5%). Regarding the place of residence, the highest proportion of participants resided in Luye Township (*n* = 61, 36.7%), followed by Chishang Township (*n* = 59, 35.5%). Regarding the source of information, the highest proportion of participants was mainly promoted by the staff at local health stations (*n* = 134, 80.7%). Regarding critical diseases, the highest proportion of participants had high blood pressure (*n* = 69, 41.6%), followed by diabetes (*n* = 48, 30.0%). Additionally, 61.4% of the participants had at least one critical chronic disease.

In terms of the difference analysis of demographic variables, this study employed *t*-tests and ANOVA methods for analysis. The results revealed that regarding the gender variable, there was a significant difference in the construct of teleophthalmology adoption between different genders (*t* = −2.822, *p* < 0.01), with females’ evaluations higher than males. However, there were no significant differences found in healthcare accessibility (*t* = −0.899, *p* > 0.05), communication quality (*t* = 0.458, *p* > 0.05), service quality (*t* = −0.831, *p* > 0.05), and health outcomes (*t* = −1.809, *p* > 0.05). Regarding the age variable, no significant differences were observed in healthcare accessibility (F = 0.849, *p* > 0.05), communication quality (F = 0.591, *p* > 0.05), teleophthalmology adoption (F = 0.549, *p* > 0.05), service quality (F = 1.041, *p* > 0.05), and health outcomes (F = 1.303, *p* > 0.05) across different age groups. Similarly, in terms of educational level, no significant differences were found in healthcare accessibility (F = 0.480, *p* > 0.05), communication quality (F = 0.721, *p* > 0.05), teleophthalmology adoption (F = 0.644, *p* > 0.05), service quality (F = 1.236, *p* > 0.05), and health outcomes (F = 0.839, *p* > 0.05) across different levels of education (Appendix B: Difference Analysis of Demographic Variables).

### 3.1. Measurement Model Assessment

The measurement model analyses included reliability, convergent validity, and discriminant validity. The present study used Cronbach’s α to assess the reliability of constructs. In the validity analysis, composite reliability (CR) and average variance extracted (AVE) were used to evaluate the convergent validity between constructs [59]. As shown in Table 2, the results of the reliability analysis revealed that the Cronbach’s α values of all the constructs other than healthcare accessibility and service quality—which was slightly lower than the value of 0.7 suggested by Nunnally [60]—were higher than 0.7. Furthermore, the confirmatory factor analysis results revealed that all constructs’ CR values exceeded 0.7. Additionally, the AVE values for all the constructs were greater than 0.5 except for the AVE value of service quality, which was slightly lower than the suggested value of 0.5. The results showed that each construct had acceptable convergent validity [58]. After confirmatory factor analysis, the goodness of fit indices for the measurement model were χ^2^/df = 1.837, GFI = 0.924, NFI = 0.934, CFI = 0.968, IFI = 0.969, RMR = 0.025, and RMSEA = 0.071. Each goodness-of-fit index met the acceptable threshold value, indicating that each construct had good validity.

Next, discriminant validity was analyzed. When the correlation coefficient between any two constructs is smaller than the square root of the AVE value for the construct, it indicates that the construct has discriminant validity [61]. Table 3 presents the results of the discriminant validity analysis. According to Fornell and Larcker [61], the discriminant validity of each construct was acceptable.

### 3.2. Structural Model Assessment

Structural model assessment reviews the goodness-of-fit indices of the structural model to determine whether there is a good fit between the research framework and real data. That is, the level of the covariance matrix for the expected research framework equals or corresponds to the level of the real covariance matrix [62,63]. Table 4 presents the model fitness of the study following the structural model analysis. Since the goodness-of-fit indices of the entire structural model were within the acceptable threshold value, the research framework of the hypothesis achieved the level of acceptance.

After analyzing the goodness-of-fit of the structural model, the hypothetical relationship was verified. The results are shown in Figure 6. The results of all hypotheses testing were supported. First, patients’ awareness of healthcare accessibility via teleophthalmology was a significant antecedent variable for both teleophthalmology adoption (β = 0.199, *p* < 0.01) and service quality (β = 0.225, *p* < 0.05). This evidence supported Hypotheses 1 and 2. Patients’ awareness of communication quality via teleophthalmology was also a significant driver of both teleophthalmology adoption (β = 0.185, *p* < 0.01) and service quality (β = 0.380, *p* < 0.001). Thus, Hypotheses 3 and 4 were supported. Finally, health outcomes were significantly affected by teleophthalmology adoption and service quality. The standardized path coefficients were 0.219 (*p* < 0.01) and 0.287 (*p* < 0.001), respectively. Therefore, Hypotheses 5 and 6 were supported. Therefore, patients’ awareness of healthcare accessibility via teleophthalmology positively affected teleophthalmology adoption and service quality. Moreover, their awareness of communication quality via teleophthalmology positively affected teleophthalmology adoption and service quality. Teleophthalmology adoption and service quality jointly affected health outcomes.

Table 5 shows the analysis of direct and indirect effects on health outcomes. Service quality had the highest direct effect, followed by teleophthalmology adoption. Also, service quality had the highest total effect, followed by teleophthalmology adoption.

## 4. Discussion

The present study identified the critical factors affecting considered healthcare outcomes via teleophthalmology in Eastern Taiwan during the COVID-19 pandemic. Generally, the average monthly cases of teleophthalmology in the five towns increased every year. This highlights the concrete effects of promoting teleophthalmology in Eastern Taiwan. The results showed that when healthcare accessibility and communication quality via teleophthalmology among the patients in the community was high, they sought medical advice via teleophthalmology and had a higher evaluation of adoption and service quality. Subsequently, people who sought medical advice via teleophthalmology had a more positive evaluation of health outcomes. Moreover, this study also indicated that teleophthalmology adoption and service quality mediate the relationship between antecedent factors (healthcare accessibility and communication quality) and health outcomes.

The findings of this study indicated that in the analysis of gender differences, there was a significant difference in teleophthalmology adoption between different genders, with females’ evaluations higher than males. This finding is similar to the results of studies by Chu et al. [64] and Wong et al. [65]. A possible explanation for this could be that female residents in remote areas face greater barriers to accessing teleophthalmology services compared to male residents. In the rural regions of Eastern Taiwan, most men have to work away from home, while women are responsible for taking care of children or the elderly, limiting their ability to visit health stations for consultations. Additionally, regarding age and educational level, there were no significant differences across all constructs, and the evaluations were generally positive (mostly above 4.0). A possible reason for this might be that before the implementation of teleophthalmology, the health stations in all five townships had already provided residents with detailed explanations and clarifications. During the process of implementing teleophthalmology, medical staff from the medical centers and health stations continually addressed patients’ concerns. Furthermore, before each consultation, personnel at the health stations would ask patients if they had any difficulties or obstacles. As a result, patient satisfaction was generally quite good.

This study found that patients’ awareness of healthcare accessibility via teleophthalmology has a significantly positive impact on teleophthalmology adoption. The results are consistent with previous studies by Haun et al. [18], Papachristou et al. [17], and Li et al. [19]. Patients’ awareness of healthcare accessibility via teleophthalmology also has a significantly positive impact on service quality. The results are similar to those of Kludacz-Alessandri et al. [33]’s study. Therefore, promoting teleophthalmology in rural areas with lower healthcare accessibility should enhance the continuance usage and service quality of telemedicine.

Patients’ awareness of communication quality via teleophthalmology is a significant antecedent variable in adopting teleophthalmology. Sabetian et al. [41] emphasized that telemedicine has been an effective physician–patient communication tool during the COVID-19 pandemic. Concurrently, communication quality is also a driving factor of service quality. Additionally, the findings of Quinton et al. [66] are similar to this finding in the present study. Therefore, enhancing the communication quality of teleophthalmology will simultaneously improve teleophthalmology adoption and service quality, as well as have a positive impact on health outcomes.

Unsurprisingly, teleophthalmology adoption and service quality simultaneously affect health outcomes. Previous studies have reported similar results [45,50,51,52]. The present study findings revealed that health outcomes are also affected by the adoption of teleophthalmology platforms and healthcare service quality. The success of teleophthalmology is determined by people’s continuous usage of teleophthalmology and the improvement in the overall service quality of teleophthalmology.

To enhance people’s awareness of healthcare accessibility via teleophthalmology, medical policies and systems should undergo innovative modifications over time to achieve the long-term benefits of teleophthalmology. The regulatory amendments include expanding the scope of telemedicine applications and insurance coverage, relaxing prescription restrictions, and increasing information security regulations [67]. It is imperative to promote important information, including the benefits of telemedicine and departments, time, and location of outpatient clinics, by strengthening health education in rural areas and using public media channels. Telemedicine should provide patients with more opportunities to obtain healthcare [68].

Determining how to use the function of digital communication in teleophthalmology to establish good and effective physician–patient communication quality and further develop physician–patient rapport is very essential. Haun et al. [18] also illustrated that the mechanisms of telemedicine have a certain impact on the users’ experience, which is a fast connection to remote specialists and establishing a harmonious relationship with remote specialists. Therefore, to achieve good communication quality in the practical promotion, telemedicine platforms and equipment must enhance the quality of technology. The robust IT platform and equipment operation and smooth network speed provide patients who seek medical advice and remote specialists a sense of presence and interaction, and it then enhances people’s willingness to revisit. In addition, the medical team also needs to focus on patients and design a convenient and time-saving process for a consultation to save their waiting time and encourage people to actively communicate with the doctor about illness, medication, treatment, and referral to achieve effective physician–patient communication.

In terms of teleophthalmology adoption, successful implementation of teleophthalmology requires several determinants. First, medical personnel at local health stations must review and understand the illness of patients who make appointments in advance. Next, fully trained personnel should communicate with and deliver suggestions from doctors at local health stations. Additionally, primary care providers at local health stations should try to shorten patients’ waiting times in clinics as much as possible. Furthermore, a set of convenient, friendly, and time-saving standard operating procedures (SOP) should be designed to integrate telemedicine with the existing physical consultation process.

To enhance the service quality of teleophthalmology, it is necessary to improve the stability of teleophthalmology equipment and platform functions, increase the output data reliability of the measuring equipment, strengthen teleophthalmology team members’ educational training and service passion, and promote telemedicine utilization. That is, the service quality of telemedicine needs to be improved simultaneously with the platform, equipment, and service personnel quality in order to comprehensively enhance the overall quality.

Regarding research limitations, the study’s participants only included people from five towns in Eastern Taiwan with teleophthalmology experience. Furthermore, convenience sampling was performed. Therefore, the extrapolation of the research results was limited because of the limitations of the research scale and sampling method. Additionally, the study only identified the patients’ perspective and failed to investigate the views of other relevant users, such as healthcare workers. Future studies can compare the evaluation of telemedicine by patients and healthcare workers. This would provide scholars and practitioners with a more comprehensive perspective and understanding and contribute more knowledge and benefits to this field.

## 5. Conclusions

This study investigated antecedents of health outcomes via teleophthalmology in Eastern Taiwan and established a research framework. The results showed that patients’ awareness of healthcare accessibility and communication quality via teleophthalmology simultaneously affected teleophthalmology adoption and service quality. Teleophthalmology adoption and service quality jointly affected health outcomes. Both teleophthalmology adoption and service quality played a mediating role. Regarding the direct and indirect effect analysis of health outcomes, service quality had the highest direct effect, followed by teleophthalmology adoption. Service quality also had the highest total effect, followed by teleophthalmology adoption. This study could serve as a reference for developing indicators for evaluation and understanding causal relationships. Future studies should follow the research framework proposed in the present study and conduct an in-depth exploration of the underlying dimensions of the four essential driving factors that affect health outcomes. It could further include other important antecedent variables and mediators (such as physician–patient trust, privacy, and security) to form a more comprehensive framework. Furthermore, future studies with relevant research designs and surveys targeting healthcare workers should provide academia and industry with a more comprehensive perspective and understanding.

## Figures and Tables

**Table 1:** Actual usage of teleophthalmology.

Township	Average Monthly Cases in 2020	Average Monthly Cases in 2021	Average Monthly Cases in 2022
Chishang	11	10.75	8.17
Luye	6.75	7.17	7.83
Guanshan	3	6.17	9.67
Haiduan	4.625	7.67	5.17
Yanping	0	0	1.5
Total	25.375	31.76	32.34

**Table 2:** Results of measurement model assessment.

Construct	Cronbach’s α	CR	AVE
Healthcare accessibility	0.68	0.70	0.54
Communication quality	0.96	0.96	0.93
Teleophthalmology adoption	0.87	0.88	0.78
Service quality	0.68	0.71	0.40
Health outcomes	0.99	0.99	0.98

CR: composite reliability; AVE: average variance extracted.

**Table 3:** Results of discriminant validity analysis.

Construct	1	2	3	4	5
1. Healthcare accessibility	(0.735)				
2. Communication quality	0.094	(0.964)			
3. Teleophthalmology adoption	0.182 *	0.179 *	(0.883)		
4. Service quality	0.143	0.333 ***	0.396 ***	(0.632)	
5. Health outcomes	0.197 *	0.161 *	0.268 ***	0.288 *	(0.990)

* *p* value < 0.05, *** *p* value < 0.001. The diagonal values are the square root of the AVE values. The other values are the correlation coefficients between corresponding constructs.

**Table 4:** Results of structural model assessment.

Goodness of Fit Index	Value	Acceptable Threshold Value	Result
χ^2^/df	1.922	<3	Good
GFI	0.920	>0.90	Good
NFI	0.932	>0.90	Good
IFI	0.966	>0.90	Good
RMR	0.034	<0.050	Good
RMSEA	0.075	<0.080	Good

GFI: goodness-of-fit index; NFI: Non-normed Fit Index; IFI: Incremental Fit Index; RMR: Root Mean Square Residual; RMSEA: Root Mean Square Error of Approximation.

**Table 5:** Results of direct and indirect effects on telemedicine outcomes.

Construct	Direct Effect	Indirect Effect	Total Effect
Healthcare accessibility	-	0.108	0.108
Communication quality	-	0.150	0.150
Telemedicine adoption	0.219	-	0.219
Service quality	0.287	-	0.287

## Data Availability

Data were generated during the study.

## Appendix A. Questionnaire Items

Healthcare accessibility:
I think that using teleophthalmology can save me time and money.
I think the duration of teleophthalmology consultations to be reasonable.
Communication quality:
I think that using teleophthalmology allows for communication with remote physicians about my condition.
I think the quality of physician-patient communication through teleophthalmology is good.
Teleophthalmology adoption:
I will continue using teleophthalmology services.
I will recommend teleophthalmology services to my friends and family.
Service quality:
I think the remote consulting physician can explain and clarify the condition clearly.
I think the teleophthalmology staff interact well with patients.
I think the audio and video quality of teleophthalmology is good.
Overall: I am satisfied with the quality of teleophthalmology services.
Health outcomes:
I think that using teleophthalmology can provide me with appropriate medical care.
I think that using teleophthalmology is beneficial for my disease treatment and health promotion.

## Appendix B. Difference Analysis of Demographic Variables

**Table A1:** Analysis of gender differences.

Construct	Category (*n*)	Mean	Standard Deviation	*t* Value	*p* Value
Healthcare accessibility	Male (62)	4.55	0.61	−0.899	0.370
	Female (104)	4.63	0.48		
Communication quality	Male (62)	4.16	0.77	0.458	0.648
	Female (104)	4.11	0.75		
Teleophthalmology adoption	Male (62)	4.25	0.84	−2.822	0.006 **
	Female (104)	4.61	0.69		
Service quality	Male (62)	4.76	0.40	−0.831	0.407
	Female (104)	4.81	0.28		
Health outcomes	Male (62)	4.21	0.72	−1.809	0.072
	Female (104)	4.42	0.74		

** *p* value < 0.01.

**Table A2:** Analysis of age differences.

Construct	Category (*n*)	Mean	Standard Deviation	F Value	*p* Value
Healthcare accessibility	≤20 (1)	5.00	0.00	0.849	0.549
	21–30 (2)	5.00	0.00		
	31–40 (4)	4.88	0.25		
	41–50 (7)	4.64	0.38		
	51–60 (28)	4.46	0.71		
	61–70 (47)	4.55	0.57		
	71–80 (46)	4.60	0.43		
	Above 80 (31)	4.69	0.48		
Communication quality	≤20 (1)	5.00	0.00	0.591	0.762
	21–30 (2)	4.00	0.00		
	31–40 (4)	4.63	0.75		
	41–50 (7)	4.14	0.75		
	51–60 (28)	4.04	0.69		
	61–70 (47)	4.07	0.80		
	71–80 (46)	4.20	0.81		
	Above 80 (31)	4.10	0.70		
Teleophthalmology adoption	≤20 (1)	5.00	0.00	0.549	0.796
	21–30 (2)	5.00	0.00		
	31–40 (4)	4.75	0.50		
	41–50 (7)	4.57	0.53		
	51–60 (28)	4.41	0.78		
	61–70 (47)	4.46	0.72		
	71–80 (46)	4.37	0.81		
	Above 80 (31)	4.60	0.85		
Service quality	≤20 (1)	5.00	0.00	1.014	0.424
	21–30 (2)	4.60	0.28		
	31–40 (4)	4.90	0.20		
	41–50 (7)	4.74	0.34		
	51–60 (28)	4.84	0.16		
	61–70 (47)	4.76	0.43		
	71–80 (46)	4.73	0.39		
	Above 80 (31)	4.89	0.14		
Health outcomes	≤20 (1)	5.00	0.00	1.303	0.252
	21–30 (2)	5.00	0.00		
	31–40 (4)	5.00	0.00		
	41–50 (7)	4.43	0.79		
	51–60 (28)	4.43	0.74		
	61–70 (47)	4.21	0.80		
	71–80 (46)	4.24	0.70		
	Above 80 (31)	4.45	0.72		

**Table A3:** Analysis of education differences.

Construct	Category (*n*)	Mean	Standard Deviation	F Value	*p* Value
Healthcare accessibility	Illiteracy (34)	4.60	0.53	0.480	0.750
	Elementary school (66)	4.59	0.39		
	Junior high school (17)	4.62	0.55		
	High school (32)	4.52	0.79		
	University level and above (17)	4.74	0.40		
Communication quality	Illiteracy (34)	3.97	0.72	0.721	0.579
	Elementary school (66)	4.23	0.80		
	Junior high school (17)	4.18	0.64		
	High school (32)	4.09	0.73		
	University level and above (17)	4.06	0.81		
Teleophthalmology adoption	Illiteracy (34)	4.47	0.90	0.644	0.632
	Elementary school (66)	4.49	0.73		
	Junior high school (17)	4.21	0.81		
	High school (32)	4.52	0.64		
	University level and above (17)	4.59	0.81		
Service quality	Illiteracy (34)	4.79	0.33	1.236	0.298
	Elementary school (66)	4.83	0.27		
	Junior high school (17)	4.71	0.32		
	High school (32)	4.82	0.27		
	University level and above (17)	4.66	0.58		
Health outcomes	Illiteracy (34)	4.47	0.75	0.839	0.502
	Elementary school (66)	4.29	0.71		
	Junior high school (17)	4.18	0.73		
	High school (32)	4.31	0.78		
	University level and above (17)	4.53	0.78		

## References

[1] World Health Organization (2023). Advice for the Public: Coronavirus Disease (COVID-19). https://www.who.int/emergencies/diseases/novel-coronavirus-2019/advice-for-public.

[2] Patel S., Hamdan S., Donahue S. (2022). Optimising telemedicine in ophthalmology during the COVID-19 pandemic. J. Telemed. Telecare.

[3] World Health Organization (2024). Implications of the COVID-19 Pandemic for Patient Safety: A Rapid Review. https://www.who.int/publications/i/item/9789240055094.

[4] Alexandra S., Handayani P.W., Azzahro F. (2021). Indonesian hospital telemedicine acceptance model: The influence of user behavior and technological dimensions. Heliyon.

[5] (2020). Centers for Disease Control. https://archive.cdc.gov/#/details?url=https://www.cdc.gov/coronavirus/2019-ncov/global-covid-19/telemedicine.html.

[6] Alboraie M., Abdalgaber M., Youssef N., Moaz I., Abdeen N., Abosheaishaa H.M., Shokry M.T., El-Raey F., Asfour S.S., Abdeldayem W.A. (2022). Healthcare providers’ perspective about the use of telemedicine in Egypt: A national survey. Int. J. Telemed. Appl..

[7] World Health Organization (2009). Telemedicine: Opportunities and developments in member states: Report on the second global survey on eHealth 2009 (Global Observatory for eHealth Series, Volume 2). Healthc. Inform. Res..

[8] Snider M.J.E., Maa A.Y., Guyton A.C., Park H., Hunt K.J., Pope C. (2022). Stakeholder perceptions affecting the implementation of teleophthalmology. BMC Health Serv. Res..

[9] Walsh L., Hong S.C., Chalakkal R.J., Ogbuehi K.C. (2021). A systematic review of current teleophthalmology services in New Zealand compared to the four comparable countries of the United Kingdom, Australia, United States of America (USA) and Canada. Clin. Ophthalmol..

[10] Ferro F., Tozzi A.E., Erba I., Dall’Oglio I., Campana A., Cecchetti C., Geremia C., Rega M.L., Tontini G., Tiozzo E. (2021). Impact of telemedicine on health outcomes in children with medical complexity: An integrative review. Eur. J. Pediatr..

[11] Anawade P.A., Sharma D., Gahane S. (2024). A comprehensive review on exploring the impact of telemedicine on healthcare accessibility. Cureus.

[12] Bhatt J., Bathija P. (2018). Ensuring access to quality health care in vulnerable communities. Acad. Med..

[13] National Center for Biotechnology Information (2024). Access to Healthcare and Disparities in Access. https://www.ncbi.nlm.nih.gov/books/NBK578537/.

[14] Oginni S.O., Opoku M.P., Nketsia W. (2022). Crisis at the intersection of four countries: Healthcare access for displaced persons in the Lake Chad Basin region. Ethnic Health.

[15] Peckham A., Pituch K.A., Maxfield M., Guest M.A., Sivanandam S., Doebbeling B.N. (2021). Aging through the time of COVID-19: A survey of self-reported healthcare access. BMC Health Serv. Res..

[16] Uwishema O., Frederiksen K.S., Correia I.F.S., Mahmoud A., Onyeaka H., Dost B. (2022). The impact of COVID-19 on patients with neurological disorders and their access to healthcare in Africa: A review of the literature. Brain Behav..

[17] Papachristou N., Vasileios R., Sarafis P., Bamidis P. (2023). Translation, cultural adaptation and pilot testing of a questionnaire measuring the factors affecting the acceptance of telemedicine by Greek cancer patients. PLoS ONE.

[18] Haun M.W., Oeljeklaus L., Hoffmann M., Tönnies J., Wensing M., Szecsenyi J., Peters-Klimm F., Krisam R., Kronsteiner D., Hartmann M. (2023). Primary care patients’ experiences of video consultations for depression and anxiety: A qualitative interview study embedded in a randomized feasibility trial. BMC Health Serv. Res..

[19] Li D., Hu Y., Pfaff H., Wang L., Deng L., Lu C., Xia S., Cheng S., Zhu X., Wu X. (2020). Determinants of patients’ intention to use the online inquiry services provided by internet hospitals: Empirical evidence from China. J. Med. Internet Res..

[20] Landi D., Ponzano M., Nicoletti C.G., Cola G., Cecchi G., Grimaldi A., Mataluni G., Mercuri N.B., Sormani M.P., Pacileo G. (2022). Patient’s point of view on the use of telemedicine in multiple sclerosis: A web-based survey. Neurol. Sci..

[21] Delone W., McLean E. (1992). Information systems success: The quest for the dependent variable. Inf. Syst. Res..

[22] Delone W., McLean E. (2003). The Delone and McLean model of information systems success: A ten-year update. J. Manag. Inf. Syst..

[23] Nuryanti Y., Hutagalung D., Nadeak M., Abadiyah S., Novitasari D. (2021). Understanding the links between system quality, information quality, service quality, and user satisfaction in the context of online learning. IJOSMAS.

[24] Uppal M.A., Ali S., Gulliver S.R. (2018). Factors determining e-learning service quality. Br. J. Educ. Technol..

[25] Parasuraman A., Zeithaml V.A., Berry L.L. (1985). A conceptual model of service quality and its implications for future research. J. Mark..

[26] Maghsoodi A.I., Saghaei A., Hafezalkotob A. (2019). Service quality measurement model integrating an extended SERVQUAL model and a hybrid decision support system. Eur. Res. Manag. Bus. Econ..

[27] Guntu M., Lin E.D., Sezgin E., Gregory M.E., Huang Y., Linwood S.L. (2022). Identifying the factors influencing patients’ telehealth visit satisfaction: Survey validation through a structural equation modeling approach. Telemed. e-Health.

[28] Rahi S., Munawar Khan M., Alghizzawi M. (2021). Factors influencing the adoption of telemedicine health services during COVID-19 pandemic crisis: An integrative research model. Enterp. Inf. Syst..

[29] Mosadeghrad A.M. (2014). Factors influencing healthcare service quality. Int. J. Health Policy Manag..

[30] Shafii M., Rafiei S., Abooee F., Bahrami M.A., Nouhi M., Lotfi F., Khanjankhani K. (2016). Assessment of service quality in teaching hospitals of Yazd University of Medical Sciences: Using multi-criteria decision making techniques. Osong Public Health Res. Perspect..

[31] Almuhanadi S., Alhammadi H., Suresh A., Al Alawi S. (2020). Assessing service quality dimensions and their effect on patients satisfaction in Bahrain primary healthcare using a modified version of the General Practice Assessment Questionnaire. Patient Prefer. Adherence.

[32] Fatehi F., Jahedi F., Tay-Kearney M.L., Kanagasingam Y. (2020). Teleophthalmology for the elderly population: A review of the literature. Int. J. Med. Inform..

[33] Kludacz-Alessandri M., Walczak R., Hawrysz L., Korneta P. (2021). The quality of medical care in the conditions of the COVID-19 pandemic, with particular emphasis on the access to primary healthcare and the effectiveness of treatment in Poland. J. Clin. Med..

[34] Unal O., Akbolat M., Amarat M. (2018). The influence of patient-physician communication on physician loyalty and hospital loyalty of the patient. Pak. J. Med. Sci..

[35] Zill J.M., Christalle E., Müller E., Härter M., Dirmaier J., Scholl I. (2014). Measurement of physician-patient communication—A systematic review. PLoS ONE.

[36] Sharkiya S.H. (2023). Quality communication can improve patient-centred health outcomes among older patients: A rapid review. BMC Health Serv. Res..

[37] Gessesse A.G., Mohammed Haile J., Woldearegay A.G. (2022). The nexus between physician-patient communication and health outcomes: Level of patient communication satisfaction and its impact on adherence in Ethiopian comprehensive specialized hospitals. Patient Prefer. Adherence.

[38] Yin S., Hu M., Chen W. (2022). Quality perceptions and choice of public health facilities: A mediation effect analysis of outpatient experience in rural China. Patient Prefer. Adherence.

[39] Hamlin M., Steingrimsson S., Cohen I., Bero V., Bar-Tl A., Adini B. (2020). Attitudes of the public to receiving medical care during emergencies through remote physician-patient communications. Int. J. Environ. Res. Public Health.

[40] Reed M., Huang J., Graetz I., Muelly E., Millman A., Lee C. (2021). Treatment and follow-up care associated with patient-scheduled primary care telemedicine and in-person visits in a large integrated health system. JAMA Netw. Open.

[41] Sabetian P.W., Ouyang V.W., Fox J.D., Jimenez A.E., Ankem H.K., Saks B.R., Maldonado D.R., Lall A.C., Domb B.G. (2023). Telemedicine: An effective tool for patient-physician communication. Orthopedics.

[42] Rehman A.U., Bashir S., Mahmood A., Karim H., Nawaz Z. (2022). Does e-shopping service quality enhance customers’ e-shopping adoption? An extended perspective of unified theory of acceptance and use of technology. PLoS ONE.

[43] Barkai G., Gadot M., Amir H., Menashe M., Shvimer-Rothschild L., Zimlichman E. (2021). Patient and clinician experience with a rapidly implemented large-scale video consultation program during COVID-19. Int. J. Qual. Health Care.

[44] Xuan Y., Guo C., Lu W. (2022). The effects of information continuity and interpersonal continuity on physician services online: Cross-sectional study. JMIR Med. Inform..

[45] Lokantari M.A., Kristaung R. (2022). Telemedicine service quality, customer satisfaction and continual usage during the COVID-19 pandemic. Budap. Int. Res. Critics Inst. J..

[46] Parmanto B., Lewis A.N., Graham K.M., Bertolet M.H. (2016). Development of the telehealth usability questionnaire (TUQ). Int. J. Telerehabil..

[47] Salam M., Bajaba S. (2021). The role of transformative healthcare technology on quality of life during the COVID-19 pandemic. J. Enabling Technol..

[48] Bokolo A.J. (2021). Exploring the adoption of telemedicine and virtual software for care of outpatients during and after COVID-19 pandemic. Ir. J. Med. Sci..

[49] Cascella M., Coluccia S., Grizzuti M., Romano M.C., Esposito G., Crispo A., Cuomo A. (2022). Satisfaction with telemedicine for cancer pain management: A model of care and cross-sectional patient satisfaction study. Curr. Oncol..

[50] Melati A.A., Haryanti D.A. (2021). Analysis of website quality on telemedicine user satisfaction in Indonesia based on measurement of end user satisfaction with the Webqual method 4.0 (Halodoc case study). Int. Res. J. Adv. Eng. Sci..

[51] Mirzaei T., Kashian N. (2020). Revisiting effective communication between patients and physicians: Cross-sectional questionnaire study comparing text-based electronic versus face-to-face communication. J. Med. Internet Res..

[52] Tantarto T., Kusnadi D., Sukandar H. (2020). Analysis of service quality towards patient satisfaction. Eur. J. Bus. Manag. Res..

[53] LeRouge C.M., Garfield M.J., Hevner A.R. (2015). Patient perspectives of telemedicine quality. Patient Prefer. Adherence.

[54] Brownsell S. (2009). Measuring the ‘success’ of telehealth interventions. J. Assist. Technol..

[55] Akter S., D‘Ambra J.G., Ray P.K.J.E.M. (2010). Service quality of mhealth platforms: Development and validation of a hierarchical model using PLS. Electron. Mark..

[56] Birkmeyer S., Wirtz B.W., Langer P.F. (2021). Determinants of mHealth success: An empirical investigation of the user perspective. Int. J. Inf. Manag..

[57] Podsakoff P.M., MacKenzie S.B., Lee J.Y., Podsakoff N.P. (2003). Common method biases in behavioral research: A critical review of the literature and recommended remedies. J. Appl. Psychol..

[58] Anderson J., Gerbing D. (1988). Structural equation modeling in practice: A review of recommended two-step approach. Psychol. Bull..

[59] Hair J., Black W., Babin B., Anderson R. (2010). Multivariate Data Dnalysis: A Global Perspective.

[60] Nunnally J.D. (1978). Psychometric Theory.

[61] Fornell C., Larcker D.F. (1981). Evaluating structural equation models with unobservable variables and measurement error. J. Mark. Res..

[62] Hair J.F., Black W.C., Babin B.J., Anderson R.E. (2014). Multivariate Data Analysis.

[63] Schermelleh-Engel K., Moosbrugger H., Müller H. (2003). Evaluating the fit of structural equation models: Tests of significance and descriptive goodness-of-fit measures. Methods Psychol. Res. Online.

[64] Chu C., Cram P., Pang A., Stamenova V., Tadrous M., Bhatia R.S. (2021). Rural Telemedicine use before and during the COVID-19 pandemic: Repeated cross-sectional study. J. Med. Internet Res..

[65] Wong H., Razvi Y., Hamid M.A., Mistry N., Filler G. (2023). Age and sex-related comparison of referral-based telemedicine service utilization during the COVID-19 pandemic in Ontario: A retrospective analysis. BMC Health Serv Res..

[66] Quinton J.K., Ong M.K., Sarkisian C., Casillas A., Vangala S., Kakani P., Han M. (2022). The impact of telemedicine on quality of care for patients with diabetes after March 2020. J. Gen. Intern. Med..

[67] KFF Health News (2020). Opportunities and Barriers for Telemedicine in the U.S. During the COVID-19 Emergency and Beyond..

[68] Cook J., Pittaoulis M., Alderfer J., Gilchrist K., Sapia M. (2023). Americans’ awareness of access changes and utilization of telehealth during COVID-19: A survey in the United States. Telemed. e-Health.

